# The evaluation of anti-angiogenic effects of Endostar on rabbit VX2 portal vein tumor thrombus using perfusion MSCT

**DOI:** 10.1186/1470-7330-14-17

**Published:** 2014-04-28

**Authors:** Guoquan Feng, Zhen Lei, Dongqing Wang, Na Xu, Qiang Wei, Dinuo Li, Jingyi Liu

**Affiliations:** 1 Radiology Department, the First Hospital of Liaoning Medical College, NO. 2, Wuduan, Renmin Street, Jinzhou 121001, China; 2 Gastroenterology Department, the First Hospital of Liaoning Medical College, NO. 2, Wuduan, Renmin Street, Jinzhou 121001, China; 3 Radiology Department, Affiliate Hospital of Jiangsu University, NO. 438, Jiefang Road, Zhenjiang 212001, China

**Keywords:** Tomography, Perfusion imaging, Portal vein tumor thrombus, VX2 tumo, Endostar, Antiangiogenic treatment, DCE-CT

## Abstract

**Background:**

There were many treatments for hepatocellular carcinoma with portal vein tumor thrombus (PVTT), in which targeted anti-angiogenic drug therapy is becoming a popular research topic. However, an objective and non-invasive method that can evaluate the treatment effects is still lacking.

**Methods:**

Eighteen New Zealand white rabbits implanted with VX2 tumor thrombus in portal vein were randomly assigned into 3 groups: Endostar, saline, or control, six in each group. Multi-slice CT (MSCT) perfusion scanning was performed to measure the differences in blood flow (TBF), tissue blood volume (TBV), and capillary permeability time the surface (PS) before and after Endostar treatment, between Endostar and saline treatment. Two weeks after treatment, both Endostar and saline groups underwent CT perfusion scan. The rabbits then were sacrificed by air embolism, and specimens of tumor thrombosis were collected. Immunohistochemistry assay was also performed to compare the expression of vascular endothelial growth factor (VEGF) in PVTT after Endostar, saline and placebo treatment.

**Results:**

In Endostar group, PVTT CT perfusion parameters (TBF, TBV, PS) significantly decreased after the treatment (p <0.05). Post-treatment PVTT CT perfusion parameters (TBF, TBV, PS) were significantly lower in Endostar group than in Saline group (p <0.05). VEGF is mainly expressed in cytoplasma. After Endostar treatment, the expression of VEGF in PVTT was markedly reduced. There was also significant difference on post-treatment VEGF protein expression measured by Immunohistochemistry assay between Endostar group and control group (p <0.05). Post-treatment PVTT CT perfusion parameters (TBF, TBV, PS) were positively correlated with VEGF protein expression in all 3 groups (r_s_ > 0, p <0.05).

**Conclusions:**

Multi-slice CT perfusion imaging can evaluate the anti-angiogenic effects of Endostar for the VX2 tumor thrombus in portal vein, and provide quantitative functional information.

## Background

Primary hepatocellular carcinoma is one of the most common gastrointestinal cancers. Having portal vein tumor thrombus (PVTT) is considered one of the main indicators for poor prognosis in liver cancer due to higher likelihood of intrahepatic and distant metastasis [1]. Current treatments for hepatocellular carcinoma with PVTT include surgery, chemotherapy, and interventional therapy, in which targeted anti-angiogenic drug therapy is becoming a popular research topic. Endostar is the generic name for recombinant human endostatin injection agent. It binds to the VEGF receptor on the tumor vascular epithelial cells, inhibits tumor angiogenesis, and blocks nutrient supply to tumor cells. However, current evaluation of treatment effect for PVTT is generally based on patients’ median survival, an objective and non-invasive method that can evaluate the treatment effects is still lacking [2,3]. Multislice spiral Computed Tomography (CT) perfusion imaging, as a functional imaging modality, quantifies perfusion and reflects focal changes of vascularization. The technique enables functional mapping of several perfusion parameters with a spatial resolution greater than that what can be achieved with other imaging techniques, and is able to indicate the changes in tissue perfusion parameters prior to the morphological alteration [4-6]. Therefore, in this study, we investigated the relationship between tumor growth and the changes in the portal vein tumor thrombus tissue perfusion parameters, analyzed the changes in PVTT CT perfusion parameters before and after Endostar treatment, to provide guidance for clinical evaluation of targeted antiangiogenic therapy for PVTT.

## Methods

### Animals

Nineteen New Zealand white rabbit, aged 4–5 months, 2.5 to 3.0 kg of body weight, 10 males and 9 females, were provided by the Laboratory Animal Center of Liaoning Medical College. The study was approved by Animal Protection Committee of Liaoning Medical College and conforms to the provisions of the Declaration of Helsinki (as revised in Tokyo 2004).

### Preparation of VX2 cell line

VX2 tumor cells were provided by Professor Lei Zhen (Department of Radiology, No. 1 Affiliated Hospital of Liaoning Medical College, Jinzhou, China). The VX2 tumor is a hypervascular tumor. The recovered tumor cells were inoculated into the muscle at rabbit’ groin area. After 14 days, a solid mass was palpable at the inoculation site. The tumor was then dissected under anesthesia. Growing tissue at the edge of the mass was collected and placed in the petri dish filled with saline. After removing necrotic and fibrous tissue, tumor tissue was cut into 1.3 mm × 1.3 mm × 4 mm size pieces, placed in saline, and extracted with 1-ml syringe connected with 14G needle (diameter of 1.54 mm) during the experiment.

### Building portal vein VX2 tumor thrombus model

The rabbits were on fasting the night before the surgery. At the day of operation, 0.4 ml of Sumianxin (the compound solution of DHE hydrochloride, Dimethylaniline and haloperidol, Changchun veterinary medicine Inc., Changchun, China) was given through intramuscular injection. After hair removal, the surgery area was disinfected with 75% alcohol. A 5 cm incision was cut at Epigastric area under the xiphoid to open the abdominal cavity and expose the liver and stomach. The liver tissue was pulled aside to find the main portal vein, and the tumor tissues were then injected into the portal vein. The injection site was pressed with gelatin sponge for about half a minute after withdrawal of the needle. Once the bleeding was stopped, chloramphenicol liquid was sprayed and the abdominal cavity was closed. Chloramphenicol was then given through intramuscular injection for three days after the surgery. The tumor tissue position and size were the same for each experimental rabbit.

### Study group assignment

Two weeks after the model development, contrast enhanced CT scan was performed at both arterial and venous phases with the followings scan parameters: 100 mA, 80 Kv, to confirm the formation of portal vein tumor thrombus. Iohexol (350 mg/ml, GE Pharmaceutical Co., Ltd., Shanghai, China) was injected into the ear vein at the rate of 0.5 ml/s with a high-pressure syringe before scanning. PVTT was confirmed by enhanced CT scan. The 18 rabbits with successful PVTT model were then randomly assigned into Endostar (15 mg/3 mL/vial, Yantai Bioengineering Co., Ltd., Yantai, China, provided by Professor Lei Zhen), Saline or control group. The rabbits assigned to control group were immediately sacrificed by air-embolus technique, and the samples of portal vein VX2 tumor thrombus were collected to measure the level of vascular endothelial growth factor (VEGF) expression prior to the Endostar treatment.

### Perfusion multi-slice spiral CT scanning

GE light speed 16-perfusion scan was performed for each rabbit. The scanning parameters were set at 100 mA, 80 kV, and center layer of the tumor at upper abdominal region was selected for dynamic scanning. Before the scanning, iohexol (350 mg/ml) was injected into the ear vein with high-pressure syringe at the dosage of 1.0 ~ 1.5 ml per kg of body weight and at the rate of 0.5 ml/s. The scan started 5 s after the injection of the contrast agent, and the exposure time was 50 s. After the scan, data were sent to ADW4.2 workstation through the LAN and analyzed using GE's Perfusion 3 liver perfusion software. The abdominal aorta was identified as the input artery, and portal vein was identified as the input vein. Pseudo-color functional imagines were generated by the computer, and the region of interest (ROI) was positioned at the PVTT to measure perfusion parameters around the PVTT: tissue blood flow (TBF, ml/100 ml/min), tissue blood volume (TBV, ml/100 ml); capillary permeability time the surface (PS, ml/100 ml/min). The ROIs along the margin of the tumor thrombus were selected based on the following criteria: the marginal zone of the tumor’s largest layer with high intensity, excluding the blood vessels, necrotic tissue and artifacts to minimize the partial volume effect on the measurements of perfusion parameters. The parameter values were measured on the mean of the parameter for each pixel inside the ROI. For each layer, at least 3 ROIs were selected, and for each rabbit, at least 2 layers were measured.

### Administration of Endostar

The standard human dose for Endostar is 15 mg/m^2^/day. After conversion from human (60 kg) to rabbit, the dose used in this study was 45 mg/m^2^/day, calculated based on the Meeh-Rubner formula: A = k × (W^2 / 3^) / 1000, where A is the body surface area (m^2^), W is the weight (g), K is a constant, 10.1 for rabbit. A rabbit with the weight of 3 kg has the body surface area of approximately 0.21 m^2^. The daily dose of Endostar for each rabbit was 9.45 mg, administrated with 100 ml saline. For Endostar and saline group, from the 3^rd^ week each rabbit was given either Endostar (9.45 mg/100 ml) or 100 ml saline by infusion through ear vein. They were then closely monitored for potential drug-related adverse reaction, which might result in slower infusion or stopping administration. At the end of 4^th^ week, GE Light Speed CT perfusion scan was performed to measure PVTT perfusion parameters (TBF, TBV, PS). After the scan, rabbits were sacrificed by air*-*embolus technique and the specimens of VX2 tumor thrombus were collected.

### Immunohistochemistry assay

Tissue specimens from all 3 groups were fixed with 10% formalin, embedded with paraffin, and cut into 5 μm thick slices. Immunohistochemistry assay was performed to determine the expression level of VEGF in tumor thrombus. Immunohistochemistry staining was conducted according to the kit instruction. The concentration of VEGF antibody was 1:300. VEGF positive staining was primarily found in the cytoplasm or cell membrane. For each slice, 5 high power fields (hpf) were randomly selected; semi-quantitative integral method [7] was used to determine the results. The final score was calculated based on the staining intensity and the percentage of positive cells among the total number of tumor cells. Staining intensity was scored as follows: 0 for colorless, 1 for amber, 2 for brown, and 3 for tan. The number of positive cells was scored as follows: 0 if less than 10% of the total cells, 1 if 10% to 20%, 2 if 21% to 50% and 3 if greater than 50%. The scores for staining intensity and positive cells were then added together to determine the final result: negative if total score was 0, weak positive (+) if 1–3, moderately positive (++) if 4–5, strongly positive (+++) if the total score was greater than or equal to 6. The evaluation was independently performed by 2 pathologists who were blinded to group assignment.

### Statistical analysis

All data were analyzed with SPSS (V17.0). The quantitative data were summarized with mean ± standard deviation, and 95% confidence interval. Perfusion parameters between two groups were compared using t-test. The level of VEGF protein expression was compared using the Wilcox on rank sum test. The relationship between PVTT CT perfusion values and VEGF protein expression was analyzed with Spearman rank correlation analysis. Analysis of variance was used to compare perfusion parameters among all three groups, and p <0.05 was defined as statistically significant.

## Results

### Tumor transplantation and pathology

**Figure 1:**
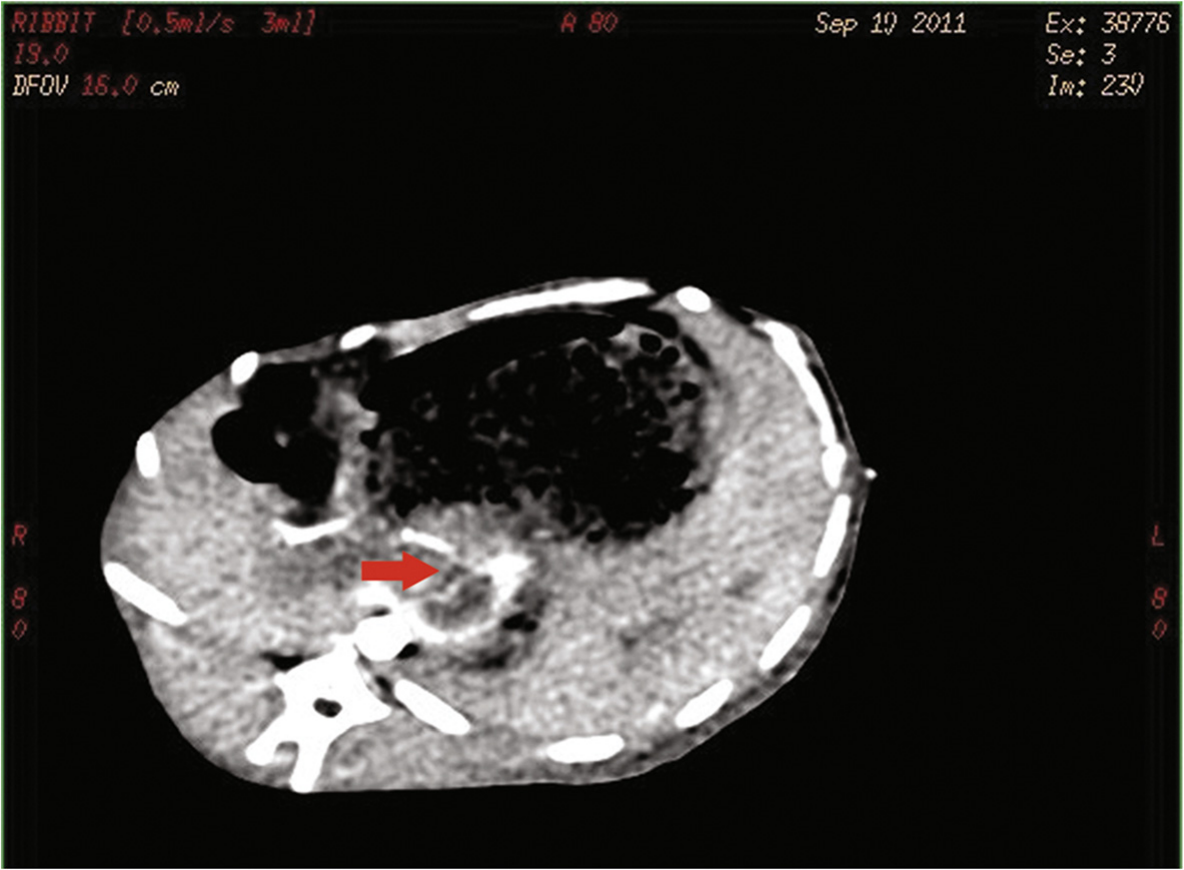
**Venous phase contrast enhanced CT scan of PVTT.** Note: the red arrow showed expansion of the portal vein, in which filling defect and mild enhancement of xenograft tumor could be observed, indicating successful tumor implantation. CT: Computed Tomography.

**Figure 2:**
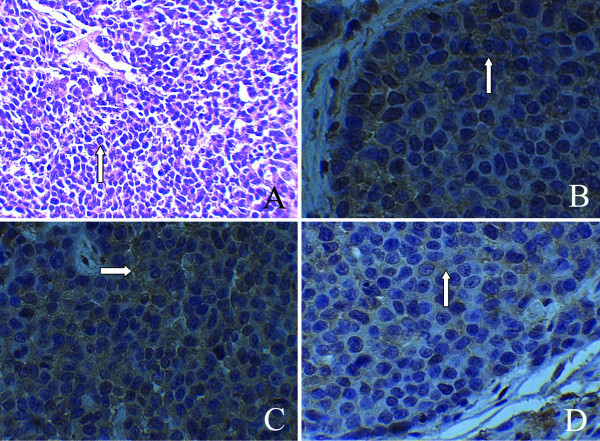
**HE staining of rabbit portal vein VX2 tumor thrombus (Figure **2**A), PVTT VEGF protein expression in control group, post-treatment Endostar and saline group (×400) (Figure **2**B, **2**C, **2**D).** Note: VEGF was mainly expressed in the cytoplasm of cancer cells and on the envelope of tumor cells. The level of VEGF protein expression was scored based on staining intensity and number of positive cells. In Figure 2-**A**, the arrow indicates cancer cells characterized by irregular shape, karyomegaly, anachromasis, and heteromorphism. Figure 2-**B** is the VEGF staining image of tumor thrombus in control group before treatment (×400). White arrow indicates VEGF expression in the cytoplasm with a score of 8 points. Figure 2-**C** is VEGF staining image of tumor thrombus in saline group (×400). White arrow indicates VEGF are widely expressed in the cytoplasm of cancer cells, some of which are deeply stained, with a score of 9 points. Figure 2-**D** is VEGF staining image of tumor thrombus in Endostar group after treatment (×400). The arrow indicates VEGF expression in cytoplasm of cancer cells, characterized by lighter stain and less positive cells, with a score of 2 points. PVTT post-treatment VEGF protein expression in Endostar group (Figure 2-**D**) was significantly lower than that in saline group (Figure 2-**C**), and also lower than pre-treatment TBF: tissue blood flow. As shown in control group (Figure 2-**B**). HE: Hematoxylin eosin. BF: blood flow. TBV: tissue blood volume. PVTT: portal vein tumor thrombus. VEGF: vascular endothelial growth factor.

The success rate of tumor transplantation was 94.4% (one rabbit died from excessive bleeding due to the penetration of the main portal vein, and another rabbit was added). Two weeks after the implantation of portal vein VX2 tumor thrombus, venous phase enhanced scan showed strip-shaped filling defect in portal vein (Figure 1). Tumor cells under low magnification demonstrated nest- or diffuse-like distribution, with infiltrative growth pattern. Marginal and interstitial areas were rich in blood vessel, showing karyomegaly and an achromas is with obvious atypia. Under high magnification, tumor cells had larger volume, round, fusiform or irregular shape, less cytoplasm, large and deeply stained nuclei, and even more obvious atypia and karyokinesis (Figure 2**-**A). Pathology results confirmed the growth of cancer cells in PVTT in all groups and there was no significant difference on pathological findings between groups in the light microscope.

### Perfusion parameters in Endostar and saline groups

**Figure 3:**
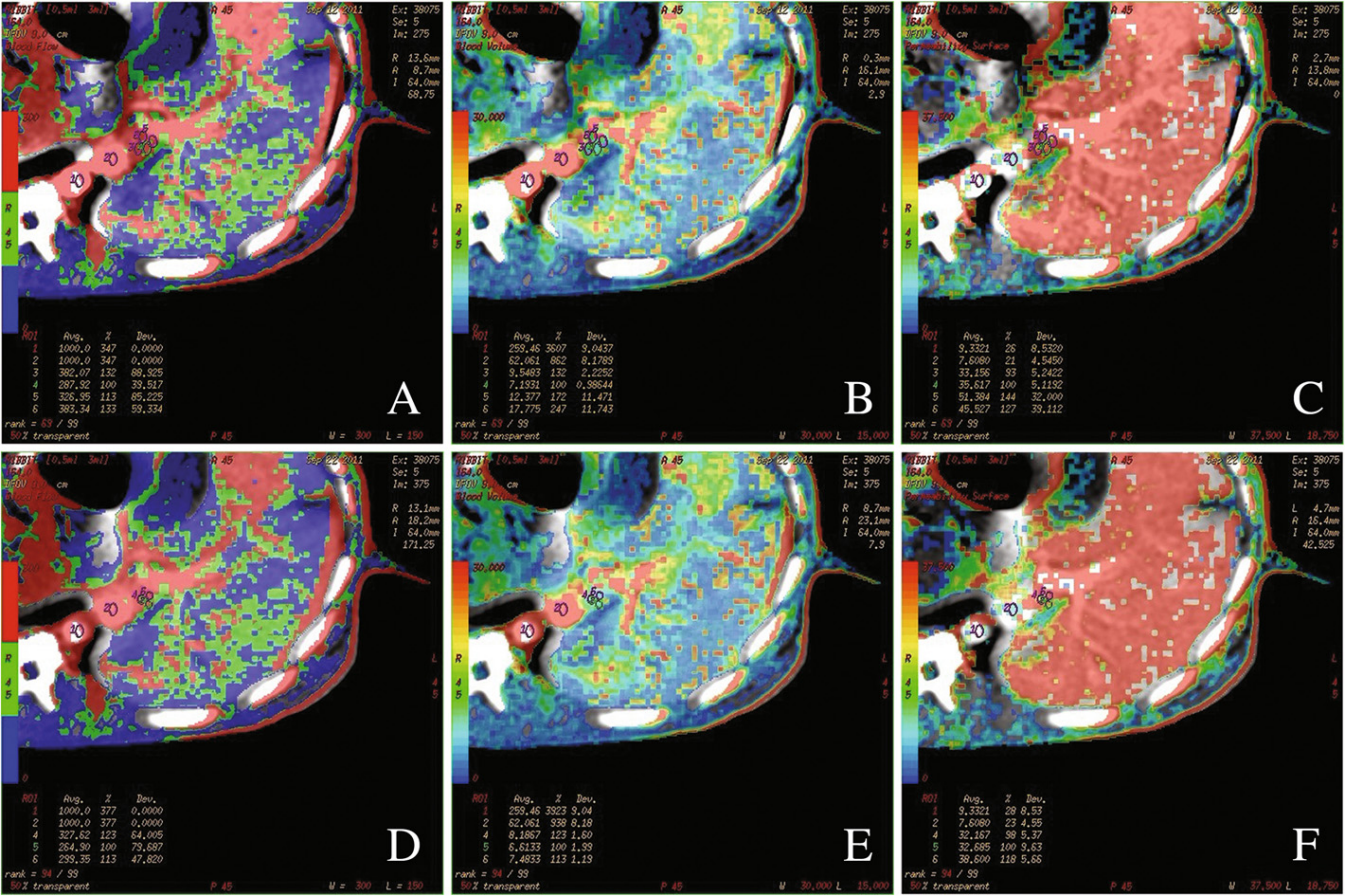
**PVTT CT perfusion images before and after Endostar treatment.** Note: PVTT Pre-treatment TBF (Figure 3-**A**), TBV (Figure 3-**B**), PS (Figure 3-**C**); PVTT post-treatment TBF (3-**D**), TBV (Figure 3-**E**), PS (Figure 3-**F**). Figure 3-**A**, 3-**D** tumor region demonstrated high blood perfusion state (red), Figure 3-**B**, 3-**E** tumor region showed high blood perfusion state (red), Figure 3-**C**, 3-**F** tumor region demonstrated high blood perfusion state (red). Post-treatment perfusion parameters were significantly lower than those at baseline. There was no notable change on tumor size.

**Figure 4:**
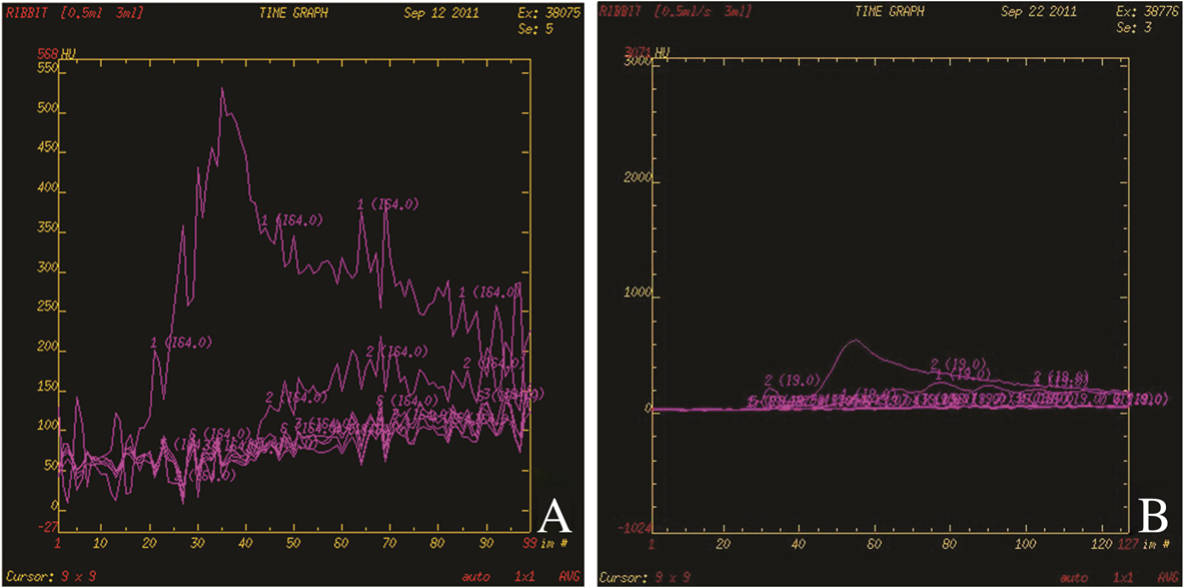
**Time-density curves for the Aorta, Portal Vein, and PVTT respectively.** Note: Figure 4**A**: Time-density curves before Endostar treatment. Curves from multiple ROIs of PVTT showed uniform pattern, suggesting the selected ROIs represented the PVTT well. Figure 4-**B**: after Endostar treatment, the peak of PVTT perfusion decreased over time when compared to before the treatment. TBF: tissue blood flow. TBV: tissue blood volume. PS: permeability surface. PVTT: portal vein tumor thrombus.

CT perfusion parameters were measured at the marginal region of the PVTT in each group. Prior to treatment (baseline), there was no statistically significant difference between 3 groups on the results of TBF, TBV and PS (p > 0.05) (Table 1). After the treatment (Table 2), in Endostar group (Figure 3**-**D, 3**-**E, 3**-**F), all three parameters significantly decreased (P <0.05) from the baseline (Figure 3**-**A, 3**-**B, 3**-**C). Before and after treatment, Time-density curve of PVTT perfusion scan was also obtained (Figure 4**-**A, 4**-**B). After Endostar treatment, peak of PVTT perfusion decreased over time. Post-treatment CT perfusion measurements were also lower (P < 0.05) in Endostar group than those in saline group (Table 3).

**Table 1:** Baseline PVTT perfusion parameters by treatment groups (n = 6)

**Parameter**	**Endostar**	**Saline**	**Control**	**F**	**p**
TBF (ml/100 ml/min)	345.07 ± 46.29	350.21 ± 20.36	347.83 ± 36.60	3.21	>0.05
TBV (ml/100 ml)	12.72 ± 2.56	11.24 ± 1.36	12.90 ± 1.90	3.60	>0.05
PS (ml/100 ml/min)	41.44 ± 5.27	42.58 ± 5.84	40.67 ± 4.38	3.52	>0.05

Note: TBF: tissue blood flow; TBV: tissue blood volume; PS: permeability surface; PVTT: portal vein tumor thrombus.

**Table 2:** PVTT perfusion parameters before and after Endostar treatment (n = 6)

**Parameters**	**Before Endostar treatment**	**After Endostar treatment**	**t**	**P value**
TBF (ml/100 ml/min)	345.07 ± 46.29	274.55 ± 26.99	17.48	0.00
TBV (ml/100 ml)	12.72 ± 2.56	7.11 ± 0.45	6.73	0.01
PS (ml/100 ml/min)	41.44 ± 5.27	32.68 ± 2.99	2.62	0.047

Note: TBF: tissue blood flow; TBV: tissue blood volume; PS: permeability surface; PVTT: portal vein tumor thrombus.

**Table 3:** PVTT perfusion parameters after treatment in Endostar and saline group (n = 6)

**Parameters**	**Post-treatment Saline group**	**Post-treatment Endostar Group**	**t**	**p**
TBF (ml/100 ml/min)	380.76 ± 13.89	274.55 ± 26.99	18.77	0.00
TBV (ml/100 ml)	20.08 ± 4.05	7.11 ± 0.45	7.35	0.01
PS (ml/100 ml/min)	47.57 ± 5.90	32.68 ± 2.99	12.18	0.00

Note: TBF: tissue blood flow; TBV: tissue blood volume; PS: permeability surface; PVTT: portal vein tumor thrombus.

### VEGF protein expression by treatment groups

VEGF protein Expression was mainly shown in the cytoplasm and envelope of tumor cells. Occasionally, VEGF protein Expression can also be seen in fibroblasts, macrophages, endothelial cells and collagen fibers in tumor interstitial (Figure 2). In Endostar group, the results of post-treatment VEGF protein Expression were 1 +++, 1 ++ and 4 +, while there were 4 +++, 2 ++ in saline group and 2 +++, 4 ++ in control group. The VEGF protein Expression from control group (Figure 4**-**B) was used to estimate the pre-treatment (baseline) level of VEGF protein Expression in Endostar group. After Endostar treatment, the PVTT VEGF protein Expression (Figure 2**-**D) was significantly reduced and lower than that in the saline group (Figure 2**-**C).

### The relationship between PVTT CT perfusion results and VEGF protein expression

There was positive correlation between PVTT CT perfusion values and VEGF protein Expression level in control, post-treatment Endostar and saline group (Table 4).

**Table 4:** **The correlation efficient (r**_**s**_**) between PVTT CT perfusion results and VEGF expression by treatment group (n = 6)**

**Group**	**TBF**	**TBV**	**PS**
Control	0.971 (p < 0.05)	0.941 (p < 0.05)	0.926 (p < 0.05)
Endostar (Post-treatment)	0.986 (p < 0.05)	0.971 (p < 0.05)	0.883 (p < 0.05)
Saline (Post-treatment)	0.986 (p < 0.05)	0.812 (p < 0.05)	0.986 (p < 0.05)

Note: TBF: tissue blood flow; TBV: tissue blood volume; PS: permeability surface; PVTT: portal vein tumor thrombus; CT: Computed Tomography; VEGF: Vascular Endothelial Growth Factor.

## Discussion

Park et al. [8] confirmed that VEGF was seen in both liver cells and HCC cells. The degree of VEGF protein Expression was associated with the level of angiogenesis and cell proliferation capacity. However, to measure the angiogenic activity using VEGF level, cancer tissue samples are required, and the test is also complicated and time-consuming. In addition, the results can only reflect the status of angiogenesis in a small portion of the tumor, rather than the entire tumor, not even mentioning the dynamic observation. Furthermore, for cancer patients who are in advanced stage and miss the chance of operation, the status of tumor angiogenesis cannot be measured with this approach due to the lack of histopathological evidence [9]. On the other hand, CT perfusion imaging as a functional imaging modality, can not only reflect the subtle structural and morphological changes of the tissues and organs, but also provide biological information through the processing of raw data.

### The change of VEGF protein Expression in PVTT before and after Endostar treatment

Our study found that post-treatment VEGF protein Expression in portal vein VX2 tumor thrombus was significantly lower in Endostar group than control group. There are several potential reasons for the reduction of VEGF protein Expression in portal vein VX2 tumor thrombus after Endostar treatment. Once reaching certain size, VX2 tumor thrombus can secrete VEGF through autocrine or paracrine signaling pathway, which acts on vascular endothelial cells to form the tumor microvasculature and support tumor growth [10]. In this study, two weeks after the transplantation of VX2 tumor into the rabbit portal vein, the tumor cells have already grown up, and showed high degree of VEGF protein expression in PVTT from immunohistochemistry assay before Endostar treatment. Endostar is a broad-spectrum anti-angiogenic drug, binds to the VEGF receptor on endothelial cells and blocks the angiogenic effect of VEGF. Endostar works directly on the microvascular endothelial cells of the tumor by affecting the endothelial growth factor receptor (VEGFR) to prevent the binding between VEGF and endothelial cell, and block the effect of VEGF. In addition, Endostar can also lower VEGF mRNA and protein expression, directly blocks the VEGF receptor signal transduction, thereby inhibits VEGF-mediated endothelial cell migration and angiogenesis [11]. After Endostar treatment, the number of microvessel within the tumor significantly decreased; consequently the oxygen and nutrient supply to the tumor tissue are reduced, leading to apoptosis of tumor cells and reduction of VEGF protein expression from tumor cells. Therefore, in this study the PVTT VEGF protein expression significantly declined after Endostar treatment.

### The change of CT perfusion parameters before and after Endostar treatment

This study found that CT perfusion parameters of PVTT significantly decreased after endostar treatment. There are several potential reasons for this observation. At the development of PVTT, tumor angiogenesis was significantly increased. Between the large numbers of immature vascular endothelial cells, the connections were loose and the gaps were larger than normal. Part of tumor vessel wall even did not have endothelial cells, leading to increased vascular permeability. Therefore, PVTT perfusion parameters were higher at baseline than after the endostar treatment [12]. The Endostar treatment may reverse certain abnormal characteristics of the tumor angiogenesis through a process named "vascular normalization" (maturation of the immature tumor blood vessels and the reduction of immature blood vessels), to reduce the permeability of tumor blood vessels. In addition, Endostar can remove less differentiated blood vessels, and retain differentiated and mature blood vessels. In this study, CT perfusion imaging demonstrated differences of tumor perfusion parameters before and after endostar treatment; the finds provided valuable experimental data for clinical evaluation the treatment effects of PVTT.

### The relationship between PVTT CT perfusion results and VEGF protein expression

In this study, PVTT CT perfusion parameters were shown positively correlated with the expression of VEGF, which may be due to the following reasons. The tumor growth, development, metastasis and prognosis are closely related to the functional status of neovascularization inside the tumor. Due to incomplete vessel walls, large gaps between adjacent vascular endothelial cells, the permeability of the new blood vessels is increased. Tumor cells can easily move in or out of the blood vessels, resulting in distant metastasis. Therefore, tumor neovascularization was an important indicator for tumor growth, metastasis, and degree of malignancy [13]. Neovascularization cannot be observed directly from the image, but can cause the changes of blood volume, perfusion volume, and capillary permeability. MSCT perfusion imaging is essentially to study the perfusion characteristics within the tumor, and can quantify the tissue blood flow, tissue blood volume, and microvascular permeability within a unit of tumor tissue, thus indirectly reflect the functional status of the tumor vasculature [14,15]. The speed of tumor growth is determined indirectly by the vascular endothelial cells, while the proliferation of endothelial cells mainly depends on VEGF. Tumor cells can synthesize and periodically secrete VEGF, which then binds with VEGF receptors on endothelial cells to promote tumor angiogenesis [16]. VEGF also has heparin binding activity, binds to receptor-2 and release various growth factors and cytokinesto stimulate tumor growth and promote endothelial cell proliferation and tumor angiogenesis [17]. When the contrast agentspass through the tumor vasculature, extravasation can be easily seen. The incomplete tumor vascular endothelium can cause the elevation in CT perfusion values. Meanwhile, due to the increase in the number of new blood vessels within the tumor, the blood supply is abundant, and the contrast agent is easy to go into the tumor tissue, resulting in the increased perfusion parameters.

## Conclusions

In summary, as a targeted anti-VEGF drug, Endostar reduces the VEGF protein expression within PVTT. MSCT, as a functional imaging modality, can obtain and analyze PVTT perfusion parameters (TBF, TBV, PS), indirectly estimate understand the extent of VEGF protein expression in PVTT, thus define the status of tumor angiogenesis and provide valuable information for evaluating the efficacy of PVTT treatment. Our study is still in the animal experimental stage. Given the high radiation exposure from CT perfusion scan, its role in the evaluation of PVTT treatment should be further investigated in clinical research.

## Competing interest

The authors declare that they have no conflict of interest.

## Authors’ contributions

GF, ZL, NX, QW, DL, JL: the conception and design of the study (or acquisition of data, or analysis and interpretation of data). GF, ZL: drafting the article or revising it critically for important intellectual content. GF, ZL, DW: final approval of the version to be submitted. All authors read and approved the final manuscript.
